# Tumor infiltrating lymphocytes against solid tumors: current trends & perspectives

**DOI:** 10.7717/peerj.20969

**Published:** 2026-06-03

**Authors:** Ze Min Liew, Yu Shyan Low, Khang Chiang Oh, Qian Yue Lee, Gee Jun Tye, Richard Bayliss, Chun-Wai Mai, Michelle Yee Mun Teo, Lionel Lian Aun In

**Affiliations:** 1 Department of Biotechnology, Faculty of Applied Sciences, UCSI University, Kuala Lumpur, Malaysia; 2 Institute for Research in Molecular Medicine (INFORMM), Universiti Sains Malaysia, Minden, Penang, Malaysia; 3 Malaysian Institute of Pharmaceuticals and Nutraceuticals, National Institutes of Biotechnology Malaysia, Halaman Bukit Gambir, Gelugor, Pinang, Malaysia; 4 Astbury Centre for Structural Molecular Biology, School of Molecular and Cellular Biology, Faculty of Biological Sciences, University of Leeds, Leeds, United Kingdom; 5 Centre for Cancer and Stem Cell Research, Institute for Research, Development and Innovation (IRDI), IMU University, Bukit Jalil, Kuala Lumpur, Malaysia

**Keywords:** Tumor infiltrating lymphocytes, Chemokines, Tumor immunology, Immunotherapy, Tumor microenvironment, Solid tumor

## Abstract

Tumor-infiltrating lymphocytes (TILs) are recognized as a key component of anticancer immunity and serve as an important prognostic factor in cancer progression. In this review, the latest updates and perspectives on the diverse populations of TILs and their roles in cancer immunity are discussed. The presence and balance between anti-tumorigenic and pro-tumorigenic immune cells in the tumor microenvironment (TME) largely determine tumor progression and fate. Thus, the properties of TILs were reviewed to provide a better insight into the roles of these immune cells within the TME. Additionally, the different factors influencing immune cell infiltration in solid tumors are also described to suggest novel immunotherapeutic approaches for improved TIL infiltration. These recent approaches are then summarised as recommendations to improve infiltration and potentially achieve better clinical outcomes. Overall, this review highlights the critical role of TILs, factors governing immune cell homing and infiltration, and strategic approaches to improve TIL infiltration.

## Introduction

**Figure 1:**
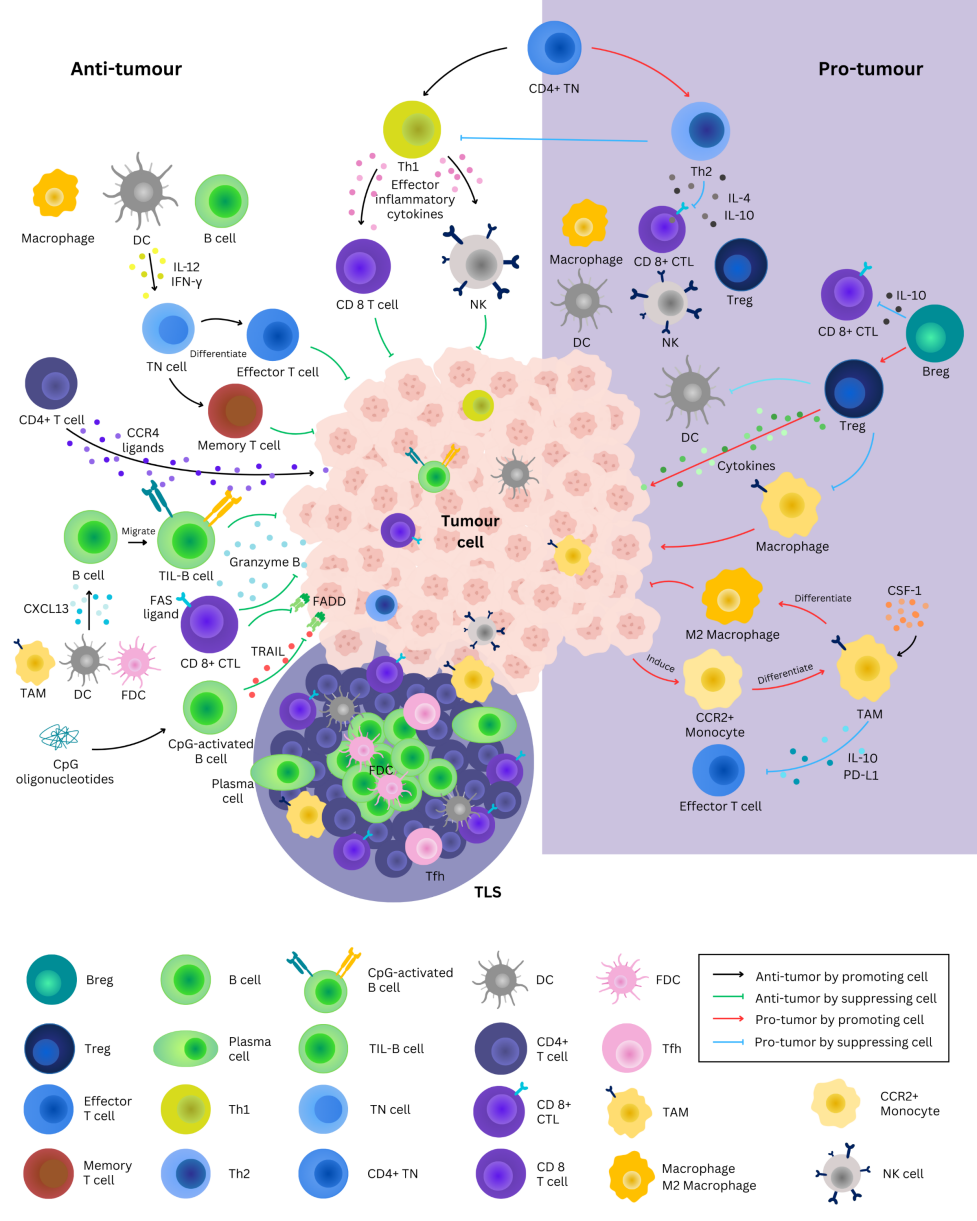
Roles of tertiary lymphoid structure and immune cells in tumor microenvironment. Tissue-resident mature DCs initiate the differentiation of naïve CD4 + T cells into distinct functional subsets, including Th1 and Th2 cells. Th1 cells secrete effector cytokines such as TNF-α, IFN-γ, IL-2 and IL12, promoting CD8 + T cells and NK cells activation and expansion. In contrast, Th2 cells secrete IL-4 and IL-10, which suppress host immune cells and promote tumor growth. CpG-activated B cells kill cancer cells *via* the TNF-related apoptosis-inducing ligand (TRAIL/Apo-2L) pathway. Cytotoxic CD8 + T cells expressed Fas ligand (FASL), which activates Fas-associated protein with death domains (FADD), leading to apoptosis of target cells. B cells migrate to the TME and form tumor-infiltrating B cells (TIL-Bs) that *via* the recruitment of CXCL13, which are secreted by immune cells such as tumor-associated macrophages (TAMs), DCs and FDCs. These TIL-Bs contribute to antigen presentation within TLS. Conversely, regulatory B cells (Breg) secrete IL-10 to negatively modulate CD8 + T cells function and suppress APCs such as macrophage and DCs. Meanwhile, DCs secrete IL-12 and IFN-γ, further promoting the differentiation of naïve T cells into effector and memory T cells. Tumor growth induces the differentiation of CCR2 + monocytes into TAMs, which secrete of IL-10 and express PD-L1 to promote effector T cells exhaustion and prevent T cell infiltration. A monocyte attractant, CSF-1 induces polarization of TAMs into immunosuppressive M2 macrophages. TILs are cluster of immune cells that resemble and infiltrate the TME generate anti-tumor immune responses. (Abbreviation: Breg, B regulatory cell; CSF-1, colony stimulating factor 1; CTL, cytotoxic T lymphocytes; DC, dendritic cell; FADD Fas-associated protein with death domains; FAS, Fas cell surface death receptor; FDC, follicular dendritic cell; Tfh, T follicular helper cell; IFN-γ, interferon gamma; IL, interleukin; NK, natural killer cell; PD-L1, programmed cell death ligand 1; Th, T helper cell; TLS, tertiary lymphoid structure; TN cell, naïve T cell; Treg, T regulatory cell).

Tumor-infiltrating lymphocytes (TILs) are immune cells that infiltrate the tumor microenvironment (TME) from the bloodstream, playing a critical role in tumor management and immune responses (Lin et al., 2020). TILs comprise of adaptive immune cells, including lymphocytes (T and B cells), macrophages and monocytes, as well as innate immune cells such as dendritic cells (DCs) and natural killer (NK) cells (Fig. 1). Collectively, TILs play a critical role in anti-tumor immunity, immunosurveillance and tumor progression. They often serve as prognostic markers in various cancers, providing valuable insight into the host immune response and therapeutic outcomes (Fanale et al., 2022). Recently, the FDA approved the first TIL-based therapy, lifileucel, for the treatment of unresectable and metastatic melanoma, highlighting the therapeutic potential of TILs in solid tumors (FDA, 2024).

The migration of TILs to the TME and tertiary lymphoid structures (TLS) begins when necrotic or apoptotic tumor cells release antigens and damage-associated molecular patterns (DAMPs), such as heat shock proteins, which are recognized by DCs through TLRs (Jhunjhunwala, Hammer & Delamarre, 2021). Activated DCs upregulate MHC I/II to present antigens to CD8^+^ and CD4^+^ T cells and express CD80/86 for full T cell activation *via* CD28 (Blander, 2023). CCR7 expressed on DCs and T cells guides their migration to lymph nodes by binding to CCL19/21 (Yan et al., 2019). This interaction directs the migration of CCR7^+^ immune cells into the T cell zones of lymph nodes, enabling the initiation of adaptive immune responses (Bandola-Simon & Roche, 2019). In the lymph nodes, naïve T cells recognize MHC–peptide complexes through their T cell receptors (TCRs) (Das & Das, 2025). The CD3ζ chain of the TCR then transmits activation signals *via* ITAM phosphorylation by the Src-family kinase Lck, initiating adaptive immune responses (Bommhardt, Schraven & Simeoni, 2019).

Tertiary lymphoid structures (TLSs) are aggregates of immune cells that form in non-lymphoid tissues at sites of chronic inflammation, including tumor sites (Schumacher & Thommen, 2022). They resemble secondary lymphoid organs like lymph nodes and create local immune responses (Sun et al., 2023). A TLS is characterized by a B cell zone, a T cell zone, high endothelial venules (HEVs), and stromal networks. The B cell zone serves as a site for B cells maturation and contains B cells, follicular dendritic cells (FDCs), and germinal center-like structures (Chen et al., 2025). On the other hand, the T cell zone is a site for T cells activation and is populated mainly by T cells and FDCs. A high density of HEVs supports the infiltration of lymphocytes. They are characterized by the expression of sialomucins and are recognized by L-selectin on naïve and memory T and B cells. Activation of the lymphotoxin β receptor (LTBR) pathway in endothelial cells promotes the differentiation of HEVs from existing vasculatures. This process can occur either with or without inhibition of vascular endothelial growth factor receptor, and is further enhanced by T cell checkpoint blockade, significantly enhancing T cell infiltration (Vella, Guelfi & Bergers, 2021). Therefore, LTBR signaling promotes the functional maturation and formation of HEVs. Activation of LTBR on endothelial cells then leads to upregulation of carbohydrate sulfotransferase 4 (CHST4), as well as CCL19, CCL21, CXCL12, and CXCL13 (Vella, Guelfi & Bergers, 2021). These chemokines play a vital role in chemotaxis, directing TILS to the TLS whilst strengthening immune surveillance and response.

Subsequently, TILs express P-selectin glycoprotein ligand-1 (PSGL-1), which mediates rolling adhesion by interacting with E-selectin expressed on endothelial cells of blood vessels (Dupas, Goetz & Osmani, 2024). To ensure firm adhesion, LFA-1 and VLA-4 on TILs also bind to ICAM-1 and VCAM-1, respectively (Murphy, 2025). These adhesion molecules are upregulated on endothelial cells in response to interferon-gamma (IFN-γ) secreted by NK cells, T helper (Th) cells, and cytotoxic T lymphocytes (CTLs). This upregulation enhances adhesion and promotes TIL extravasation into the TME. Once adhesion is secured, matrix metalloproteinases (MMPs) facilitate the migration of TILs into the interstitial region, either through paracellular routes (between cells) or *via* transcellular pathways (through cells). Within the interstitial space, CXCR4 on TILs detects CXCL12 signals, while CXCL9 and CXCL10 produced by tumor cells further support their retention within TLSs, promoting sustained immune surveillance and anti-tumor activity (Gorchs et al., 2022).

While these mechanisms enable immune cell entry and positioning, their efficiency is frequently undermined by tumor-intrinsic and microenvironmental constraints that attenuate signal transduction and effector competence. In solid tumors, dysregulation of chemokine axes, stromal and extracellular matrix (ECM)–mediated physical barriers, and profound T cell exhaustion collectively impair immune cell infiltration and limit cytotoxic activity. These clinical challenges underscore the need for targeted TIL strategies that enhance immune cell trafficking, persistence, and effector function within the TME. Emerging approaches include the use of immune checkpoint inhibitors to reinvigorate exhausted T cells, immunocytokines to enhance local immune activation, and adoptive cell therapies such as CAR-engineered and gene-edited T cells which are designed to improve tumor homing and persistence. In parallel, the application of oncolytic viruses and ECM-modulating agents also aim to remodel the TME to facilitate immune cell access. Therefore, by bridging the mechanistic understanding of TIL biology and the mechanisms governing tumor infiltration, this review highlights the factors governing immune cell infiltration and key opportunities to clinically improve patient outcomes through TIL-based immunotherapy.

## Review Methodology

This review was conducted through a comprehensive literature search across multiple scientific databases, including Google Scholar, ScienceDirect and Nature Publishing Group, among other reputable sources. The primary search terms included tumor-infiltrating lymphocytes, challenges of T cell infiltration, T cell exhaustion, and chemokines. The search strategy was further expanded to encompass related subtopics such as immune checkpoint inhibitors, cell engineering, chimeric antigen receptors, tumor microenvironment, oncolytic viruses, and the extracellular matrix. Combinations of these keywords, with or without Boolean operators, were employed to ensure a broad and inclusive search scope. Abbreviations of chemokines, their respective receptors, and the names of distinct tumor infiltrating lymphocyte subsets were also incorporated into the search. Titles and abstracts of the retrieved articles were screened for relevance, and selected studies were systematically integrated into the review. This iterative process prioritized works published within the past five years to maintain the review’s timeliness, accuracy and relevance.

### Properties of tumor infiltrating lymphocytes

Different subsets of tumor infiltrating lymphocytes (TILs) exert distinct functions that target tumors through both direct and indirect mechanisms (Bremnes et al., 2016; Garutti et al., 2024). TILs serve as markers of disease outcomes in various cancers, including melanoma, head and neck, breast, bladder, urothelial, ovarian, colorectal, renal, prostatic and lung cancers. In most cases, a high level of TIL infiltration is associated with a positive clinical outcome (Table 1). Their presence within the TME reflects cancer progression and prognosis indications as the host’s anti-tumor immune response takes place in the TME (Lim et al., 2023).

### TIL antigen presentation in TME

Antigen presenting cells (APCs) are specialized immune cells that present processed antigens to T lymphocytes, facilitating their infiltration into tissues (Lee-Chang & Lesniak, 2023). Among APCs, tissue-resident mature DCs are the most potent, as they activate and direct naïve antigen-specific T cells, including CD4^+^ and CD8^+^ T cells, which mediate the elimination of diseased cells (Suarez et al., 2020). However, recent studies showed that DCs express high levels of programmed death-ligand 1 (PD-L1), which weakens the activation of T cells and inhibits anti-tumor activity. This occurs through the binding of PD-1 molecules on effector T cells to CD80/CD86 and PD-L1 on APCs or tumor cells, leading to T cell dysfunction (Zhao et al., 2022). Therefore, the precise role of DCs in tumor immunity remains unclear, as they have been associated with both favorable and unfavorable prognosis (Yu et al., 2022).

In addition to DCs, B cells also migrate to the TME and differentiate into tumor-infiltrating B cells (TIL-Bs) *via* the recruitment of CXCL13 (Xue et al., 2024). CXCL13, also known as B cell chemoattractant-1, is secreted by immune cells such as DCs, FDCs and tumor-associated macrophages (TAMs), and contributes to tumor immunity within the TME. TIL-Bs regulate tumor growth by antibody production, cytokines secretion and antigens presentation (Chang et al., 2022). TIL-Bs possess high expression of MHC I and MHC II molecules, thus rendering them the capability to present antigens (Rastogi et al., 2022). Notably, TIL-Bs are more efficient than tumor-associated DCs in antigen presentation due to their selective presentation of cognate antigens *via* surface immunoglobulin (Ig) molecules. This efficiency correlates positively with overall survival (OS) in several solid tumors including non-small cell lung cancer (NSCLC) and breast cancer (Bruno et al., 2017). In addition to the role of TIL-Bs as APCs, they have also been reported to secrete C-C motif chemokines ligand 22 and 17 (CCL22 and CCL17), which recruit CD4^+^ T cells to the TME (Rastogi et al., 2022).

**Table 1:** Types of common tumor infiltrating lymphocytes and its roles in TME.

**TIL type**	**Subtype**	**Function in TME**	**Effect on tumor**	**Solid cancer type**	**Reference**
T cells	CD8	Directly kill tumour cells	Anti-tumorigenic	Lung cancer	Geng et al. (2015)
			Anti-tumorigenic	Oral squamous cell carcinoma	Shimizu et al. (2019)
			Anti-tumorigenic	Pancreatic adenocarcinoma	Ino et al. (2013)
			Anti-tumorigenic	Breast cancer	Sun, Ke & Li (2022)
	CD4	Activate immune cells like B-lymphocytes, cytotoxic T cells and non-immune cells (Luckheeram et al., 2012)	Anti-tumorigenic	Lung cancer	Geng et al. (2015)
			Anti-tumorigenic	Pancreatic adenocarcinoma	Ino et al. (2013)
			Anti-tumorigenic	Cervical cancer	Wang et al. (2019)
	Th1 CD4		Anti-tumorigenic	Colorectal cancer	Tosolini et al. (2011)
	CD45RO	Mount a quicker and higher magnitude immune response to reinfections (Künzli & Masopust, 2023)	Anti-tumorigenic	Gastric cancer	Lee et al. (2008)
	Treg	Inhibit immune cell activation, proliferation and effector functions, including cytokine production (Ohkura & Sakaguchi, 2020)	Pro-tumorigenic	Lung cancer	Geng et al. (2015)
			Pro-tumorigenic	Pancreatic adenocarcinoma	Ino et al. (2013)
B cells	CD20	Regulate the activation and proliferation of B cell (Pavlasova & Mraz, 2020)	Anti-tumorigenic	Oropharyngeal cancer	Young et al. (2024)
	Breg	Suppress immune response (Catalán et al., 2021)	Pro-tumorigenic	Hepatocellular carcinoma	Garnelo et al. (2017)
NK cells	CD56	Serve as innate immune cells that lyses virally infected and tumourigenic cells without antigen sensitisation (Mace, 2022)	Anti-tumorigenic	Neuroblastoma	Melaiu et al. (2020)
			Pro-tumorigenic	Cervical cancer	Wang et al. (2019)
Dendritic cells	CD1a	Act as antigen presenting cells (Song et al., 2018)	Anti-tumorigenic	Diffuse Large B cell Lymphomas	Chang et al. (2007)
	CD83		Anti-tumorigenic		
M2 Macrophages	CD163	Regulate inflammatory reactions and promote pro-tumoural functions by driving tumour growth and metastasis (Su et al., 2020)	Pro-tumorigenic	Cervical cancer	Wang et al. (2019)
	CD204		Pro-tumorigenic		
Mast cells	Inactivated	Induce angiogenesis and promote tissue growth (Maciel, Moura & Hermine, 2015; Varricchi et al., 2017)	Pro-tumorigenic	Cervical cancer	Wang et al. (2019)

### CD4^+^ TIL subsets

In the TME, exposed to chronic antigen stimulation, a subset of CD4^+^ TILs directly kill autologous tumor cells in an MHC class II-dependent manner (Hall et al., 2023). These cytotoxic CD4^+^ T cells express effector molecules such as granzyme B, granzyme K, perforin, granulysin, and NKG7, and transcriptionally resemble CD8^+^ T cells (Oh et al., 2020). Naïve CD4^+^ T cells differentiate into diverse functional subsets in response to context-dependent signals. The two major subsets of Th cells, Th1 and Th2 cells exhibit distinct cytokine profiles and mechanisms of action (Zhao et al., 2019). Th1 cells produce tumor necrosis factor-alpha (TNF-α), IFN-γ, IL-2 and IL-12, which enhance the expansion and effector functions of CD8^+^ T cells and NK cells. Additionally, IFN-γ also inhibits the differentiation of Th2 and Th17 cells (Zhou et al., 2022). In contrast, Th2 cells secrete IL-4 and IL-10, which generally promote tumor growth by inhibiting host anti-tumor immunity. However, Th2 cells have also been shown to inhibit the progression of colon and pancreatic cancers through the generation of Fas and Fas ligand (FasL), hypersecretion of IL-5, and recruitment of macrophage and eosinophil, which mediate tumor cell killing (Jacenik, Karagiannidis & Beswick, 2022). Despite the distinct mechanisms and balance between Th1 and Th2 subsets, a dominant Th1 response is nevertheless clinically associated with better clinical outcomes, including increased survival rate and lower cancer reoccurrence (Zhao et al., 2019).

Th17 cells, a subset of CD4^+^ Th cells, primarily produce IL-17, which induces the expression of other cytokines such as IL-6, IL-21, IL-22, IFN-γ and IL-1. The role of Th17 cells in cancer is paradoxical, as they can elicit both pro-tumor and anti-tumor effects (Marques et al., 2021). Th17 cells have been reported to promote angiogenesis (Anvar et al., 2024) and initiate malignant transformation, a function that depends on Krüppel-like factor 6, T cell-specific T-box transcription factor and IFN-γ (Fesneau et al., 2024). Conversely, Th17 cells have been shown to enhance stemness, reduce exhaustion, and exert superior tumor infiltration capabilities, likely mediated by IFN-γ and IFN regulatory factor 7-dependent type I IFN responses (Lei et al., 2024).

### Anti-tumor effector TILs

NK cells serve as early anti-tumor responders by recognizing transformed cells through activating and inhibitory receptors, including the detection of missing MHC-I, elimination of cancer cells *via* perforin–granzyme release, death receptor pathways, and antibody-dependent cellular cytotoxicity (ADCC) through FcγRIIIa (Wu et al., 2020; Yu et al., 2022; Fantini, Arlen & Tsang, 2023). They also secrete IFN-γ and TNF-α to support adaptive immunity (Liu et al., 2021). Similarly, M1 macrophages contribute to tumor control through direct cytotoxicity and ADCC, with TNF-α-induced upregulation of indoleamine 2, 3-dioxygenase 1 (IDO1) expression and nitrite production, activation of caspase-3 and poly (ADP-ribose) polymerase (PARP-1) cleavage for mitochondria-dependent apoptosis (Guerra et al., 2017). Additionally, they also generate reactive oxygen species (ROS) and nitric oxide *via* inducible nitric oxide synthase (iNOS) and secrete IL-12 to enhance anti-tumor immune responses (Pan et al., 2020).

Cytotoxic CD8^+^ T cells are the most potent effector cells, inducing target cell death through the release of cytotoxic granules containing granzymes, perforin, cathepsin C, and granulysin, which form pores in the target cell membrane. Additionally, FasL expressed on CD8^+^ T cells activates Fas-associated protein with death domain (FADD), leading to caspase activation, endonuclease induction and DNA fragmentation in target cells (Raskov et al., 2020). While classical killing depends on TCR recognition of neoantigen–MHC-I complexes (Durgeau et al., 2018), CD8^+^ T cells can also eliminate MHC-I–deficient tumor cells *via* NKG2D engagement with stress-induced NKG2D ligands (Lerner et al., 2023), thus enabling effective cytotoxicity despite immune evasion.

Tumor-infiltrating B cells (TIL-Bs) further enhance anti-tumor immunity through antigen presentation, cytokine secretion, and antibody production, which enables them to mediate ADCC, antibody-dependent cellular phagocytosis, and complement-dependent cytotoxicity. TIL-B differentiation into tumor-reactive plasma cells ultimately leads to the production of tumor-reactive IgG1 antibodies within the TME, thereby supporting humoral immunity and tertiary lymphoid structure formation (Meylan et al., 2022; Chen, Chu & Liu, 2024; Kim et al., 2025). Beyond antibody-mediated effects, TIL-Bs can also directly exert their cytotoxic effects through the secretion of granzyme B (Willsmore et al., 2021) or activation of TNF-related apoptosis-inducing ligand (TRAIL/Apo-2L) pathways (Gupta et al., 2022), and their abundance consistently correlates with improved patient survival across multiple cancer types (Xu-Monette et al., 2023).

### Pro-tumor effector TILs

The dominant mechanism of TIL-B mediated immunosuppression is the secretion of IL-10 by B regulatory cells (Bregs). IL-10 negatively modulates the function of CD8^+^ T cells, promotes the development of T regulatory cells (Tregs) and suppresses the activity of APCs, such as DCs and macrophages, thus suppressing effector T cells activity in TME. Moreover, Bregs can secrete IL-35 and TGF-β that also contribute to immune suppression by expanding Tregs and Bregs and enhancing anti-inflammatory cytokine. IgA-producing Bregs have also been identified within the TME. Studies have shown that the frequency and absolute number of IL-10 producing Bregs are elevated in NSCLC patients, correlating positively with increased Tregs, reduced survival, and advanced clinical stages (Bruno et al., 2017). Bregs have also been shown to suppress CD8^+^ T cell proliferation and inhibit the expression of proinflammatory cytokines in both CD4^+^ and CD8^+^ T cells (Mohib et al., 2020). Furthermore, B cells can promote angiogenesis and cause circulating immune complex deposition that promotes carcinogenesis (Rastogi et al., 2022).

Tregs are essential for maintaining immune tolerance and prevent excessive inflammation. However, in cancer, these immunosuppressive mechanisms suppress anti-tumor immunity and hinders immune surveillance against cancer development (Su et al., 2022). Elevated levels of both type 1 T regulatory cells (Tr1) and Bregs have been shown to impair tumor immunity and are associated with a decreased Disease Activity Score-28 (DAS28), erythrocyte sedimentation rate, and C-reactive protein levels (Hsieh et al., 2023). Even though these markers are commonly applied in autoimmune disease settings, similar immune dysregulation in cancer patients contributes to poorer survival and can negatively influence overall health status and treatment outcomes (Hannibal et al., 2024; Singh & Das, 2025). Bregs can further exacerbate immunosuppression by inducing the conversion of conventional CD4^+^ T cells into Tregs, thereby reinforcing tumor immune tolerance and reducing overall patient survival (De Matteis et al., 2018). Tregs further encourage the formation of an immunosuppressive TME by releasing inhibitory cytokines and interacting with stromal components, including fibroblasts and endothelial cells, thereby promoting cancer cell survival (Zhang et al., 2024).

Another sub-population of immune cells within the TME are TAMs which comprise a heterogenous cell population accounting for up to 50% of the cellular composition in solid tumors (Li et al., 2022a). Derived primarily from C-C chemokine receptor 2 (CCR2)^+^ monocytes, TAMs exhibit functional plasticity and can polarize into either M1 or M2 phenotypes, which contributes to the structural organization of the TME. While M1 macrophages exert anti-tumor effects, the TME predominantly favors M2-like TAMs, which support tumor growth, immune evasion, angiogenesis and metastasis. Factors such as C5a and colony-stimulating factor 1 enhance TAM recruitment and drive M2 polarization (Ghebremedhin et al., 2024). M2-like TAMs suppress cytotoxic T-cell infiltration and function by secreting IL-10, expressing immune checkpoint molecules such as PD-L1 (Li et al., 2022a), and inhibiting CD8^+^ T-cell motility, collectively contributing to poor clinical outcomes (Peranzoni et al., 2018; Xu et al., 2022).

## Factors that Affect Tumor Infiltration

### T cell exhaustion

T cell exhaustion is a major barrier to effective anticancer immunity, characterized by reduced cytolytic ability, impaired cytokine production and reduced proliferative capacity. Exhausted T cells express high levels of inhibitory receptors such as PD-1, T cell immunoglobulin and mucin domain 3 (TIM-3), cytotoxic T-lymphocyte associated protein 4 (CTLA-4), lymphocyte-activation gene 3 (LAG-3) and T cell immunoreceptor with immunoglobulin and immunoreceptor tyrosine-based inhibitory motif domains (TIGIT), which collectively suppress T cell function (Chin et al., 2022; Jenkins et al., 2023). Chronic T cell activation drives upregulation of these receptors to prevent excessive T cells activation. Interaction of an inhibitory receptor with its corresponding ligands initiates T cell exhaustion by reducing TCR signaling, including CD28 co-signaling (He & Xu, 2020) and CD4^+^ T cells activation (Maruhashi et al., 2018), ultimately reducing proinflammatory cytokine and cytolytic protein expression. In addition, key downstream pathways such as PI3K/AKT/mTOR can be suppressed by PD-1 and CTLA-4, thereby disrupting metabolic processes—including glycolysis and oxidative phosphorylation that are essential for T-cell expansion and effector function (Wang et al., 2024).

Multiple immunosuppressive cells, including Tregs and TAMs, contribute to T-cell exhaustion, as described in pro-tumor effector TILs. These cells secrete cytokines such as IL-10, IL-35, and TGF-β, which upregulate immune checkpoints and drive TIL exhaustion (McRitchie & Akkaya, 2022; Kzhyshkowska, Shen & Larionova, 2024). Several studies have since targeted these cytokines or their receptors to block immunosuppressive signaling and restore antitumor T-cell activity (Yi et al., 2022; Zhou et al., 2023). Interestingly, IL-10 has demonstrated context-dependent effects in cancer immunity, with reports showing that it can enhance CD8^+^ TIL cytotoxicity by promoting its activation and expansion (Emmerich et al., 2012; Wang et al., 2016).

Although immune checkpoints normally serve to limit the overactivation of T cells, their high expression in the TME weakens intrinsic anti-cancer immune response. Elevated inhibitory receptor expression has been correlated with poor cancer prognosis (Ishikawa et al., 2020; Wang et al., 2025a). The expression of the key proinflammatory cytokine, IFN-γ is reduced in exhausted T cells, resulting in impaired immune cell recruitment in tumor (Brunell et al., 2023). IFN-γ increases TILs activity by increasing Th1 response and CD8^+^ T cells differentiation, suppressing Tregs and promoting T cell recruitment (Castro et al., 2018). Thus, immunotherapy such as PD-1/PD-L1 and CTLA4 inhibitors have been used to restore T cell exhaustion and increase TIL activity (Teo et al., 2022; Ausejo-Mauleon et al., 2023; Principe et al., 2024). However, the therapeutic efficacy of these inhibitors varies depending on patients’ immune profiles and resistance mechanisms, which will be further discussed in a later section.

### Chemokine-mediated migration

The most common mechanism guiding the directional migration of TILs is the establishment of chemotactic gradients. These gradients are generated by cytokines and chemokines released by granulocytes, including neutrophils, eosinophils, and basophils (Van der Woude et al., 2017). However, some of these cells can also exert pro-tumor functions, such as recruiting additional immunosuppressive populations (Ghaffari & Rezaei, 2023; Wang et al., 2025b), indicating that both the timing and type of chemoattractant are critical for effectively directing anti-tumor immune cells. As T cells approach the tumor stroma, they migrate intratumorally along chemokine-bound ECM structures, adopting elongated morphologies that facilitate navigation through the TME and ultimately contribute to tumor control (Tan et al., 2015).

Different immune cell subsets express distinct chemokine receptors, which are classified into four groups based on their structural motifs: C-C motif chemokine receptor (CCR), C-X-C motif chemokine receptor (CXCR), C-X3-C motif chemokine receptor (CX3CR), and X-C motif chemokine receptor (XCR). They are all examples of G protein coupled receptors (GPCRs), differentiated by the position of the conserved cysteine disulphide bonds pairs. Although all four chemokine receptor groups share similar signaling pathways, they are activated by distinct chemokines. These chemokines are categorized into four types: CC ligand (CCL), CXC ligand (CXCL), CX3C ligand (CX3CL), and XC ligand (XCL).

Chemokines activate their receptors through heterotrimeric G proteins, which consists of α, β and γ subunits (Wu, 2005). Upon receptor activation, the G protein α subunit exchanges GDP for GTP and dissociates from the βγ subunit. Both subunits interact with downstream effectors, ultimately regulating cell migration by influencing cell polarity. The α subunits are divided into four groups, each with distinct functions: G_α_*i*, G_α_*s*, G_α_*q*,  and G_α_12 (Syrovatkina et al., 2016). G_α_*i* inhibits adenylyl cyclase (Campbell & Smrcka, 2018), G_α_*s* stimulates adenylyl cyclase (Wettschureck & Offermanns, 2005), G_α_*q* activates phospholipase Cβ (Kamato et al., 2015) and G_α_12 activates Rho guanine nucleotide exchange factors (GEFs) (Siehler, 2009). The Gβγ subunit activates phosphoinositide 3-kinases γ (PI3Kγ) (Stephens et al., 1997), phospholipase C β (PLCβ) (Camps et al., 1992), p21-activated kinase (PAK) (Wang et al., 1999), and phosphatidylinositol-3,4,5-trisphosphate dependent Rac exchange factor 1 (P-REX 1) (Welch et al., 2002).

Rho GEF–dependent activation of RhoA promotes actin polymerization and contractility, generating the structural forces needed for immune cell movement (O’Connor & Chen, 2013). PI3K-derived phosphatidylinositol (3,4,5)-trisphosphate, establishes cell polarity for gradient sensing, while PLCβ-driven calcium signaling supports migration in response to chemokines such as CXCL12. Additional regulators, including PAK1, which facilitates the expression of CCR7 and L-selectin in activated CD4^+^ T cell through the c-Jun N-terminal kinase (JNK) pathway (Dios-Esponera et al., 2019), and P-REX1, which promotes macrophage migration by activating Rac (Wang et al., 2008), further enhance immune cell motility. Together, these pathways demonstrate how chemokine receptor signaling coordinates cytoskeletal remodeling and directional movement, enabling effective immune cell infiltration into tumors.

### Gene expression and chemokine levels affecting TILs

Chemokine receptors are mainly expressed on immune cells and mediate immune cell migration by responding to chemokine gradients. However, due to the complexity of the immune system where various cells produce different sets of chemokines, the outcomes of chemokine-based therapies can sometimes be unpredictable. As a result, even in tumors with high TILs infiltration, the presence of pro-tumor immune cells may reduce overall survival (OS) in cancer patients (Tables 2 and 3).

**Table 2:** Anti-tumor role of chemokine receptors and ligands in tumor infiltrating lymphocyte.

**Chemokine**	**Receptors**	**Recruited immune cells**	**Prognosis**	**Reference**
CCL5	CCR1, CCR3	CD8^+^, activated CD4^+^ memory T cells, activated NK cells and M1 macrophages	Increase OS, HR = 0.39	Araujo et al. (2018)
CCL11	CCR3	Tregs, memory CD4^+^ T cells, CD8^+^ T cell, neutrophils, M1 macrophages, and Tfh cells	Increase OS, HR = 0.87	Chen et al. (2024)
CCL16	CCR2	mast cells, gamma delta T cells, monocytes, and naive CD4^+^ T cells	Increase OS, *P* < 0.05	Li et al. (2024)
CCL17	CCR4	Resting and activated CD4 memory T cells resting, resting NK cells, resting and activated DCs, CD8^+^ T cells, PCs, mast cells and monocytes	Increase OS, HR = 0.59 at stage 1 cancer	Ye et al. (2022)
CXCL9	CXCR3	B cells, CD8^+^ T cells and CD4^+^ T cells	Increase OS, HR = 0.75	Liang et al. (2021)
CXCL11	CXCR3	CD8^+^ T cells and activated NK cells	Increase OS, *P* = 0.0053	Cao et al. (2021)
CXCL13	CXCR5	CD4^+^ and CD8^+^ T cells, CD20^+^ B cells, and CD38^+^ PCs	Increase OS, *P* < 0.05	Ukita et al. (2022)
CXCL16	CXCR6	CD4^+^ and CD8^+^ T cells	Increase OS, *P* = 0.041	Hojo et al. (2007)
CXCL17	CXCR8	CD4^+^ T cells and B cells	Increase OS, HR = 0.7	Wang et al. (2022)
CX3CL1	CX3CR1	CD1a^+^ DCs and CD8^+^ T cells	Increase OS, *P* < 0.001	Park, Lee & Yoon (2012)

**Notes.**

Abbreviation HR hazard ratio OS overall survival

**Table 3:** Pro-tumor role of chemokine receptors and ligands in tumor infiltrating lymphocyte.

**Chemokine**	**Receptors**	**Recruited immune cells**	**Prognosis**	**Reference**
CCL1	CCR8	Tregs	Reduce OS, *P* = 0.149	Kuehnemuth et al. (2018)
CCL2	CCR2	M2 macrophages	Reduce OS, HR = 1.744 ± 0.056	Kim et al. (2024)
CCL4	CCR3	M0 macrophages	Reduce OS, HR = 1.444	Zhang et al. (2021)
CCL15	CCR1, CCR3	CD14^+^ monocytes	Reduce OS, HR = 1.650 ± 0.246	Liu et al. (2019)
CCL19	CCR7	Tregs	Reduce OS, *P* = 0.017	Xu et al. (2023)
CCL20	CCR6	Monocytes, neutrophils, TAMs, Th1 cells, Tregs and exhausted T cells	Reduce OS, HR = 1.865 ± 0.600	Zhai et al. (2023)
CCL21	CCR7	Plasmacytoid DCs	Reduce OS, *P* < 0.05	Zhao et al. (2024)
CCL22	CCR4	Tregs	Reduce OS, *P*<0.0001	Li et al. (2013)
CCL23	CCR3	Exhausted CD8^+^ T cells and macrophages	Reduce OS, HR=1.66	Kamat, Krishnan & Dorigo (2022)
CCL28	CCR3	Foxp3^+^ Tregs	Reduce OS, *P* = 0.0112	Yan et al. (2021)
CXCL3	CXCR2	Macrophages and neutrophils	Reduce OS, *P* = 0.022	Lv et al. (2021)
CXCL5	CXCR1, CXCR2	Activated NK cells and resting mast cells	Reduce OS, *P* = 0.045	Nie et al. (2021)
CXCL7	CXCR2	Macrophages and neutrophils	Reduce OS, HR = 1.3	Wang et al. (2022)
CXCL8	CXCR1	Macrophages, neutrophils and Th1 cells	Reduce OS, HR = 2.05	Shen et al. (2022)
CXCL10	CXCR3	CD4^+^ and CD8^+^ cells, macrophages, neutrophils, dendritic cells, B cells	Reduce OS, HR = 1.1	Wang et al. (2022)
CXCL12	CXCR4	Foxp3^+^ T cells	Reduce OS, *P* = 0.0476	Zhang et al. (2019)
XCL2	XCR1	CD8^+^ T cells, M1 macrophages	Reduce OS, *P* < 0.05	Chen et al. (2023)

**Notes.**

Abbreviation HR hazard ratio OS overall survival

Among chemokine receptors, CXCR3, CCR5 and CXCR4 show the highest expression in CD4^+^ and CD8^+^ T cells (Löfroos et al., 2017). Higher CXCR3 expression is associated with increased DC infiltration and enhanced CD4^+^ and CD8^+^ TIL infiltration, which in turn correlates with improved OS in gastric cancer (Chen et al., 2018). In serous ovarian cancer tissues, production of CXCL9 and CXCL10—ligands of CXCR3—promotes infiltration of CD56^+^ NK cells, CD3^+^ T cells and FOXP3^+^ T cells. The release of these ligands is mediated by IFN-γ and TNF-α through the JAK and NF-κB signaling pathways (Bronger et al., 2016).

Single cell RNA sequencing has shown that in an exhausted environment, T cells and NK cells express CXCR6 and CCL3. These environments have higher cytotoxic potential and contain more chemotactic attractants, including CCL3, CCL4, CCL5, and CXCL9. In addition, a high frequency of CD4^+^ T cells is observed in tumor regions enriched with CXCL9, CCL22 and CXCL13. Similarly, CD8^+^ T cells infiltrate areas with high CXCL9 and CXCL10 expression. Notably, CXCL13 expression serves as an indicator of immune exhaustion and is important for TLS formation. Most CXCL13^+^ T cells appear in mature TLS, where they play a role in attracting B cells (Tietscher et al., 2023).

## Strategies for Enhanced Infiltration in Solid Tumors

**Figure 2:**
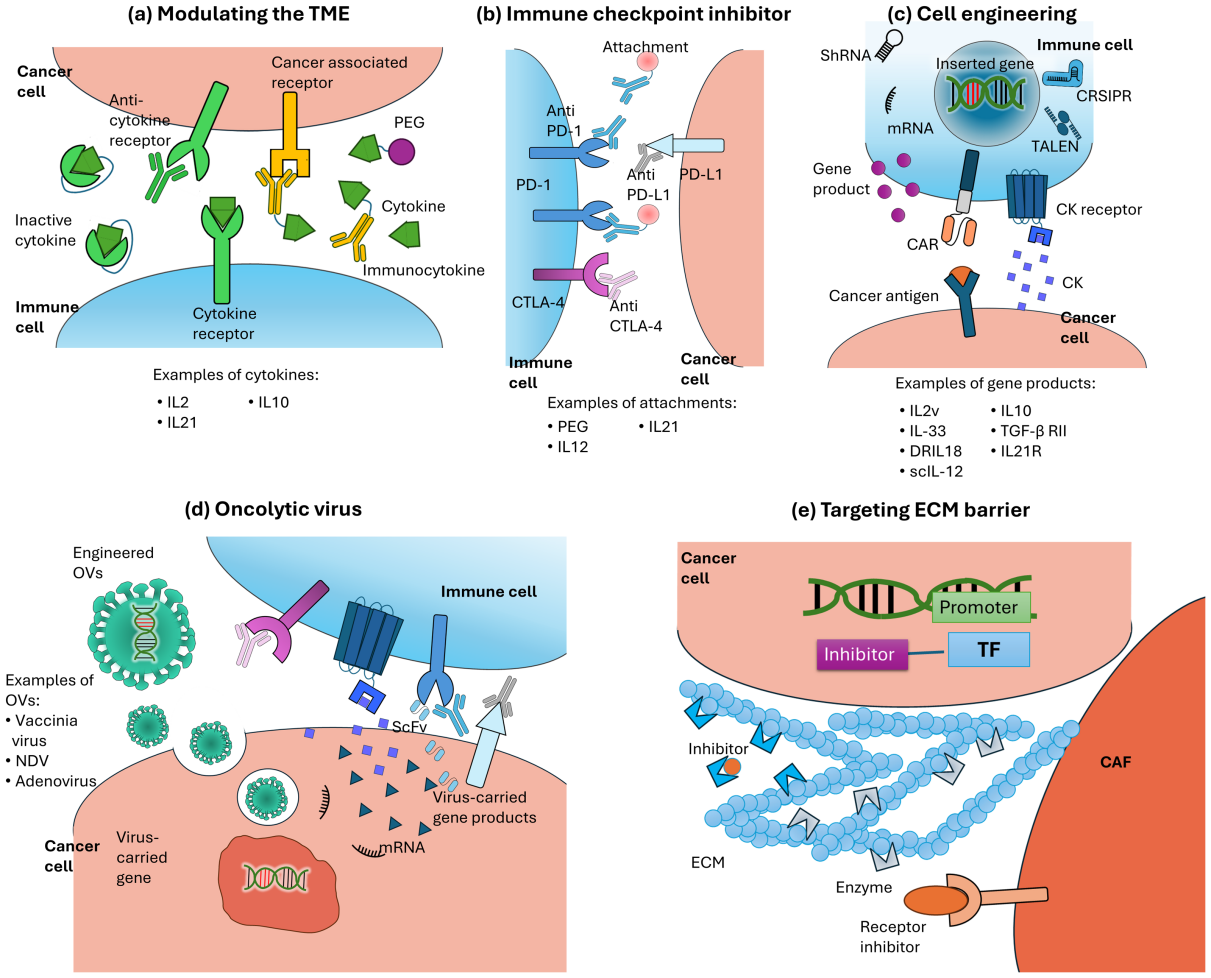
Strategies to enhance migration and survival of tumor infiltrating lymphocyte in solid tumor. (A) Cytokines can stimulate immune responses to enhance anti-tumor activity and may be engineered for improved stability and targeted delivery. Additionally, antibodies are used to inhibit unwanted signaling. (B) Immune checkpoint inhibitors (ICIs) target inhibitory receptor and their ligands to prevent T cell exhaustion. The antibodies can be engineered with functional attachment to improve therapeutic efficacy and drug delivery. (C) Cellular expression profiles can be modified through genetic or non-genetic approaches to express effector molecules/receptor for cancer antigen recognition (CAR receptor), effector molecules or shRNAs for immune cell survival, and factors that promote migration and recruitment *via* chemotaxis. Gene-editing tools such as CRISPR and TALEN can also be used to disrupt inhibitory pathways. (D) Oncolytic viruses can be engineered to carry additional genes that enhance immune cell infiltration into tumors. When combined with ICIs, they further prevent T cell exhaustion and show better anti-tumor activity. (E) Enzymes and enzyme inhibitors are used to reconstruct the ECM, targeting signaling pathways involved in tumor progression and ECM stiffening. (Abbreviations: CAF, cancer-associated fibroblast; CAR, chimeric antigen receptor; CK, chemokine; CTLA-4, cytotoxic T-lymphocyte-associated protein 4; DRIL18, decoy-resistant interleukin-18; ECM, extracellular matrix; IL, interleukin; NDV, Newcastle disease virus; OV, oncolytic virus; PD-1, Programmed cell death protein 1; PD-L1, programmed cell death ligand 1; PEG, polyethyleneglycol; ScFv, single-chain variable fragment; scIL-12, single-chaininterleukin-12; TF, transcription factor; TGF-β RII, transforming growth factor beta receptor II.).

An immunosuppressive TME represents a major barrier to immune cell infiltration in solid tumors, prompting the development of various immunotherapeutic strategies to enhance anti-tumor response. Recent studies suggest that approaches combining tumor targeting, TME remodeling and rejuvenating exhausted immune cells may increase the number of intratumoral TILs (Fig. 2). Notably, these combinatorial immunotherapeutic approaches often show synergistic effects, further improving therapeutic outcomes.

### Modulating the TME

Cytokines with immunostimulatory effects have been employed to reshape the TME and enhance antitumor immunity. For instance, cytokine-based therapies inhibit immunosuppressive cells and promote recruitment of TILs (Matsueda et al., 2024). On the contrary, recombinant cytokine therapies often exhibit short half-life and may cause on-target off-tumor toxicity (Bonati & Tang, 2021). To overcome these limitations, cytokines are engineered with improved bioavailability and armed with tumor targeting abilities.

Some researchers have combined antibodies with cytokines to generate immunocytokines that enhance targeted cytokine delivery while limiting systemic toxicity. Tumor necrosis-targeting human IgG1 (NHS)-IL-2 is the first tumor-targeting cytokine to enter human phase 1 clinical trial (Strauss et al., 2019). The IL-2 molecule was fused with NHS76, an antibody that targets tumor necrotic regions (Fallon et al., 2014). This design prolongs the cytokine’s half-life compared to recombinant IL12 *in vivo*. NHS-IL-2 increases the frequency of activated NKT cells in peripheral blood and increases immune cell infiltration into tumors (Fallon et al., 2014; Strauss et al., 2019). Similarly, Herceptin-murine IL-12, an immunocytokine targeting human epidermal growth factor receptor 2 (HER2) increases CD4^+^ and CD8^+^ T cells infiltration in murine colorectal tumor models. Additionally, the extend of T cell infiltration correlates with the intratumoral penetration of immunocytokines, which in turn is dependent on antibody-binding kinetics (Jung et al., 2022). In another strategy, conjugation of a tumor-homing peptide, Cys-Asn-Gly-Arg-Cys (NGR) to TNF-α targets CD13 on tumor vasculature. This modification promoted endothelial cell activation and facilitated CD8^+^ T cell infiltration in melanoma models (Calcinotto et al., 2012).

In addition, cytokines can be engineered to improve bioavailability and target specificity. Another modified IL-2 (sumIL-2) was engineered with preferential binding to CD122 over CD25 to reduce its effect on Tregs. Fusion of sumIL-2 to an anti-human EGFR (Erb- sumIL-2) not only extended its *in vivo* half-life but also increased the intratumoral ratio of CD8^+^/Treg, CD8^+^/CD4^+^ and CD4^+^/Treg (Sun et al., 2019). Similarly, the EGFR-targeting immunocytokine IL-21–Erb reduced the proportion of exhausted PD-1^+^ CD8^+^ T cells and increased IFN-γ^+^ in CD8^+^ T cells within the tumor (Deng et al., 2020). Fusion of IL-12 to extracellular domain of its receptor (IL12-Fc) has also been used to increase the half-life of the cytokine and reduce systemic toxicity. The fusion protein incorporates an MMP14 substrate sequence, thus enabling IL-12 release specifically within TME, where MMPs are overexpressed. IL-12-Fc treatment increases the abundance of IFN-γ^+^ CD8^+^ T cells in MC38 tumor and suppresses tumor growth in tumor-bearing mice (Xue et al., 2022). Similar approach involving the fusion of IL-2 to CD25 was reported to selectively reduce T cell exhaustion and increase CD8^+^ T cells infiltration in colorectal cancer and melanoma models (LaPorte et al., 2023).

Various strategies using antibodies or other factors to disrupt immunosuppressive pathways within TME have also shown promise. An insulin-like growth factor (IGF)–blocking antibody increased cytotoxic T cell infiltration in pancreatic cancer models, potentially *via* upregulation of CXCL9 and CXCL10 following IGF blockade (Freeman et al., 2024). Similarly, a TGFβRII antibody inhibited Smad2 signaling, reducing tumor cell migration and metastasis while enhancing CD8^+^ T cell infiltration and limiting Treg-mediated immunosuppression in breast and pancreatic cancer models (Zhong et al., 2010). Targeting chemokine axes has also shown efficacy: anti-CCR8 antibodies depleted intratumoral CCR8^+^ Tregs and induced tumor regression in colorectal and breast cancers (Nagira et al., 2023), while CCR2 inhibition reduced myeloid-derived suppressor cell infiltration into gliomas and enhanced T cell activation when combined with anti-PD-1 therapy (Flores-Toro et al., 2020). Likewise, blockade of CCL20 suppressed head and neck squamous cell carcinoma growth by limiting Treg infiltration and differentiation (Rutihinda et al., 2023). Cytokine modulation approaches have also been explored; PEGylation of IL-10 (pegilodecakin) extended its half-life and reduced TGF-β levels, alleviated CD8^+^ T cell exhaustion, and increased intratumoral phospho-STAT3^+^ CD8^+^ T cells, contributing to improved antitumor responses (Mumm et al., 2011; Naing et al., 2018).

### Immune checkpoint inhibitors

Immune checkpoint inhibitors (ICIs) are a class of immunotherapies designed to reduce T cell exhaustion and enhance both anti-tumor activity and tumor infiltration by targeting the immune checkpoint receptor or its ligand (Hoo, Siak & In, 2019). It is well known that patients often exhibit low response rates, and many develop resistance to ICI therapy, which encourages the use of combinatorial therapy over monotherapy (Li & Sun, 2024).

### Resistance towards ICIs

One key factor underlying the variability in patients’ responses to ICI treatments is the heterogeneity of their tumor and immune features, such as the concentration of PD-1 and tumor mutational burden (Di Federico et al., 2024). Tumors with low immunogenicity or minimal inflammation often termed “cold” tumors which fail to respond to immunomodulatory cues, resulting in primary resistance to ICIs (Liu, Li & Li, 2024). In addition, low expression of NAD(P) dependent 3-beta-hydroxysteroid dehydrogenase has been shown to upregulate TGF-β1 and inhibit IFN-γ, contributing to resistance against ICIs (Xie et al., 2023a). Prolonged ICI monotherapy may also lead to T-cell desensitization, limiting therapeutic benefit. As a result, current strategies increasingly focus on combinatorial approaches, antibody conjugates, and advanced delivery systems to improve treatment outcomes.

### ICI combination therapies

A combination of anti-PD-1 and anti-CTLA-4 therapies has demonstrated a synergistic effect on exhausted TILs, and especially on high PD-1 expression TILs (Kim et al., 2021). A recent study reported that mutation of the *STK11* gene is associated with low PD-L1 level in tumors, which possibly contributes to poor response to PD-L1 inhibitor therapy. However, this effect was abrogated with the addition of anti-CTLA treatment. Dual ICI therapy increased CD4^+^ T cells and improved the Th1/Treg ratio within the tumor (Skoulidis et al., 2024). Sequential treatment with anti-CTLA followed by anti-PD1 antibodies remodeled the TME by enhancing anti-tumor cytokine production and downregulating angiogenesis-related genes. This treatment also promotes the activation and accumulation of CD4^+^ and CD8^+^ T cells, significantly improving patient responses to anti-PD1 treatment (Ma et al., 2024). Regorafenib suppresses TGF-β1 by increasing the expression of NAD(P) dependent 3-beta-hydroxysteroid dehydrogenase. When combined with anti-PD-L1, this treatment further increased the number of Granzyme B^+^ cells and reduced Treg infiltration (Xie et al., 2023a).

### Drug delivery systems for ICIs

Polymeric particles have become a popular solution for targeted drug delivery to retain ICIs at the tumor site and minimize systemic ICI toxicity (Kiaie et al., 2023). A truncated anti-PD-L1 peptide chain conjugated with PEG formed a micelle-like nanoparticle, which remained inactive until cleaved by MMP-2 within the TME. This design ensures that the therapeutic peptide is released specifically within MMP-2–rich tumor regions, preventing off-target exposure. Upon activation, the peptide retained its ability to bind PD-L1 and increases IFN-γ expression and CD8^+^ T cell infiltration in the TME (Zhao et al., 2025). Mannose-modified macrophage-derived microparticles (Man-MPs) loaded with metformin (Met@Man-MPs) increased the efficacy of anti-PD-1 therapy by repolarizing M2-like TAM to M1 phenotype and promoting tumor collagen degradation. When combined with anti-PD-1 antibodies, Met@Man-MPs increased antibody penetration and accumulation in the tumor, along with greater infiltration of activated CD8^+^ T cells (Wei et al., 2021).

### ICI-cytokine conjugates

Some researchers are focusing on anti-PD-1 antibody as a homing antibody to deliver therapeutic agents directly to target immune cells. This approach enables targeted activation of tumor-reactive T cells while limiting systemic toxicity. For example, anti-PD-1 antibody conjugated with a mutated IL-12 shows selectively binding towards PD-1^+^ CD8^+^ TILs, thus reducing systemic IL-12 toxicity. In addition, this conjugated antibody promotes IFN-γ and granzyme B expression, particularly in PD-1^+^ Tim3^+^ CD8^+^ T cells population (Zou et al., 2024). Similarly, anti-PD-1 antibody conjugated with IL-21 binds to tumor specific T cells and promotes both systemic and intratumoral expansion of OT-1 cells derived from ACT (Li et al., 2021).

### Cell engineering

The most commonly engineered immune cells for adoptive cell therapy (ACT) are chimeric antigen receptor (CAR) immune cells, which include CAR T cells, CAR B cells and CAR NK cells. One of the main disadvantages of CAR immune cells is their limited ability to penetrate solid tumors (Looi et al., 2024). Thus, additional functional proteins have been introduced to enhance their antitumoral effects against solid tumors.

Several studies have demonstrated that engineering CAR T cells to overexpress chemokine receptors can enhance tumor infiltration, although this improvement remains dependent on the level of chemokine ligand expression within the TME. For example, CXCR2-expressing CAR T cells exhibit superior trafficking and intratumoral expansion in tumors with high CXCL2 expression, leading to enhanced tumor control (Liu et al., 2020). Similarly, another study showed that CXCR6-engineered T cells enhanced tumor infiltration through interaction with tumor-derived CXCL16, resulting in prolonged survival in a pancreatic cancer model (Lesch et al., 2021).

Additional engineering strategies have been explored to enhance CAR immune cell survivability and reduce exhaustion. For instance, HER2-specific CAR T cells engineered to downregulate the three major inhibitory receptors: PD-1, LAG-3 and TIM-3, *via* shRNA insertion exhibited improved infiltration capabilities. These cells secreted higher levels of IFN-γ, showed upregulation of CD56. High expression of CD56 in CAR T cells leads to enhanced anti-tumor activity and resistance to apoptosis (Zou et al., 2019). In another study, IL-10-expressing HER2 CAR T cells promoted cell proliferation and effector molecule expression by enhancing OXPHOS in MPC-dependent and antigen-dependent manner, resulting in improved tumor killing in colon, breast, and melanoma models; inhibition of OXPHOS abrogated these effects (Zhao et al., 2024). CAR NK cells modified with TGF-β RII/IL21R can convert immunosuppressive TGF-β signals into IL21R-pSTAT3 activation, reducing the frequency of PD-1^+^ TIM3^+^ exhausted NK cells and increasing cytotoxicity *via* the JAK-pSTAT3 pathway (Ren et al., 2025).

Some researchers focus on engineering immune cells to provide additional functions, such as increased proliferation and tumor homing. For example, adoptive transfer of T cells engineered with PD1d/IL-2 Vand PD1d/IL-33 led to an increased population of TCF1^−^ TILs. This ACT approach induced a synthetic T cell state marked by TOX^−^ PD-1^+^ Gzmc^+^ expression, in which the TILs’ function was not limited by the expression of inhibitory receptors. In addition, some TILs show a PD-1^−^ phenotype with low levels of granzyme C expression. IL-2v contributed to the emergence of a novel T cell state by evading cell exhaustion through TOX pathway (Corria-Osorio et al., 2023). In melanoma, Pmel-1 T cells targeting gp100 show limited natural efficacy, prompting efforts to improve their function (Overwijk et al., 2003). Electroporation of CD8^+^ Pmel-1 T cells with DRIL18 and scIL-12 mRNA promoted adhesion to E-selectin molecules and increased cellular metabolism. A single intratumoral injection of these engineered Pmel-1 T cells show bilateral therapeutic effects, demonstrating the synergistic anti-tumor activity of both genes (Olivera et al., 2023). PD-1 knockout in tumor-reactive Pmel-1 T cells *via* TALEN-mediated gene editing improved persistence and infiltration at tumor sites, while also increasing GZMB and IFN-γ expression (Menger et al., 2016). Alternatively, PD-1 disruption through CRISPR-Cas9 engineering yielded only minimal effects on TIL cytotoxicity and reactivity markers (Chamberlain et al., 2022).

In addition to genetic modification, chemical and nanotechnological methods for cell engineering are also being explored. M1 macrophage modified with an aptamer-cholesterol conjugate represents a non-genetic modified option for targeted cell therapy. The aptamer provides tumor-specific targeting, significantly enhancing the expression of proinflammatory cytokines on the macrophage surface. These engineered M1 macrophages show improved anti-tumor activity by reducing tumor density and promoting necrosis in breast cancer models (Yang et al., 2024). Another study reported that M1 macrophages engulfed single-walled carbon nanotube composites encapsulating ferric oxide, rendering the cells ‘magnetic’ and enabling magnet-guided tumor targeting in breast cancer models (Zhang et al., 2023).

### Oncolytic virus and combinatorial therapy

Oncolytic viruses have been used as a new way to improve the efficacy of immunomodulatory agents, as they can selectively infect tumor cells. Initially, oncolytic viruses were primarily used to induce tumor cell lysis. Upon infection, tumor cells begin to express viral proteins which ultimately lead to cell lysis through apoptosis or necrosis (Lin, Shen & Liang, 2023). However, the low replication rate of viruses, lack of virus entry proteins, and host immune responses towards oncolytic viruses reduce their efficiency in lysing tumor cells (Martinez-Quintanilla et al., 2019). Hence, more recent studies have focused on enhancing the immune system’s ability to kill tumors through combination therapies. Combining ICIs with viral vectors for transgene expression has become a new trend to promote TILs infiltration. The presence of cytokines can direct the infiltration of anti-tumor immune cells, increase immune cell persistence, and enhance cancer cell killing. The anti-tumor effect can be further enhanced through combination therapies with ICIs and ACT and can target not only the injected tumor but also distant tumors (Ju et al., 2022).

Vaccinia virus (VV), an oncolytic virus carrying the IL-2 gene, promotes the CD8^+^ T cells activation and infiltration in MC38 tumor cells (Feist et al., 2021). Similar results were observed using an adenovirus engineered to express IL-2 (Havunen et al., 2017). Notably, the number of exhausted PD-1^+^ Tim3^+^ CD8^+^ TILs reduces when treated with VVs (Feist et al., 2021). High expression of caspase 3 was observed in exhausted CD8^+^ T cells after transfection with VVs suggests that these cells undergo apoptosis (DePeaux et al., 2024). High levels of TGFβ in the TME disrupt IFN-γ signaling and hinder the efficacy of VV treatment. Hence, a recombinant VV expressing a TGFβ inhibitor has been developed to mitigate tumor resistance to viral treatment. This results in a higher percentage of CD4^+^ and CD8^+^T cells in the virus-transfected tumor group, with further enhancement observed upon combination with anti-PD-1 therapy (DePeaux et al., 2023).

A recent study on animal ACT demonstrated that adjunct with IL-2 transgene-carried adenovirus increased granzyme B expression among CD4^+^ T cells, CD8^+^ T cells and Tregs (Quixabeira et al., 2023). Recombinant Newcastle disease viruses (NDV) express the ICOS ligand induced high expression of ICOS in melanoma and promoted TIL infiltration. Mice bearing melanoma with high ICOS expression allowed stimulation of Th cell expansion and reduced PD-1 expression on CD8^+^ T cell (Zamarin et al., 2017).

Combinatorial therapy with oncolytic viruses and ICIs has increased CD4^+^ and CD8^+^ T cells infiltration (Marek et al., 2023). This has been achieved either by administering oncolytic viruses and ICIs separately or by engineering oncolytic viruses to carry ICI genes. Recombinant NDV were engineered to express an anti-CD28-IL12 recombinant protein. When combined with systemic anti-CTLA4 treatment, this therapy resulted in complete tumor response and a significant increase in intratumoral CD8^+^ T cell populations, which exhibited high CD28 expression (Vijayakumar, McCroskery & Palese, 2020). CT26 colon carcinoma tumors were infected with a recombinant oncolytic virus that made them produce extracellular TIGIT-Fc, which binds to CD155 on the tumor. This treatment increased CD8^+^ T cell infiltration and reduced the number of Tregs in the tumor (Xie et al., 2023b). Expression of humanized PD-1 single chain fragment variable by a recombinant oncolytic virus promoted the expansion of activated CD8^+^ T cell and reduced both immunosuppressive cells and exhausted CD8^+^ T cells within the tumor. When combined with anti-TIM-3 or anti-CTLA-4 therapy, this treatment elicited T cell activation and significantly enhanced anti-tumor efficacy (Ju et al., 2022).

A recombinant oncolytic virus encoding the CXCL10 gene promoted lymphocyte migration toward cancer cells through chemotaxis. High expression of CXCL10 within the TME increased infiltration of CD4^+^ and CD8^+^ T cells. Combination therapy with anti-PD-1 antibodies significantly upregulated the expression of IFN-γ and granzyme and enhanced tumor killing, depending on the number of CXCR3^+^ T cells (Li et al., 2022b). Oncolytic herpes simplex virus HF10 increased PD-L1 expression in tumor cells. Interestingly, CD8^+^ TILs expressed low levels of inhibitory receptors, including PD-1, TIGIT and TIM3 (Eissa et al., 2021).

The presence of a high number of infiltrated virus-specific bystander T cells has been observed in the TME upon oncolytic virus treatment. These bystander T cells with killing ability can be directed to the tumor by introducing an oncolytic virus expressing a bystander T cell epitope. A high number of CD4^+^ bystander T cells contribute to tumor cytotoxicity in a granzyme B-dependent manner. In addition, the recombinant oncolytic viruses can promote DCs responses, resulting in improved epitope separation and better tumor control (Chen, Chu & Liu, 2024). Another study shows oncolytic viruses can promote the expansion of virus-specific CD8^+^ T cells, and when engineered to express tumor-associated antigens, they can shift the CD8^+^ T cell population shifts towards tumor specificity. In addition, the administration of ICIs prior oncolytic virus treatment further increases the expansion of virus-specific CD8^+^ T cells (Webb et al., 2024). These studies open up a new frontier for further enhancing the efficacy and selectivity of immunotherapies in combination with appropriately engineered oncolytic viruses.

### Targeting ECM barriers

ECM stiffening, which refers to increased ECM rigidity, is a key barrier to effective immunotherapy and promotes tumor progression. In a high-stiffness ECM milieu, cancer cells receive signals that activate EMT, characterized by increased N-cadherin and decreased E-cadherin expression. This shift enhances the cancer cell motility and invasiveness (Zhang & Zhang, 2025). Furthermore, the stiff ECM forms a physical barrier around that restricts immune cells infiltration (Chung, Xie & Suk, 2021) and limits the penetration of chemotherapeutics and ICIs (Jurj et al., 2022). Therefore, disruption of the ECM might result in enhanced immune cell infiltration and improved drug delivery within the TME.

Pegvorhyaluronidase alfa (PVHA), a recombinant glycosidase, is also capable of reducing ECM stiffness by catalyzing the hydrolysis of hyaluronic acid. PVHA improves the penetration of anti-PD-L1 antibodies in breast tumors thereby enhancing the infiltration of CD8^+^ T cells, CD4^+^ T cells and NK cells, ultimately leading to tumor regression. Notably, PVHA also ameliorates immune cell infiltration in an anti–PD-L1-resistant cancer model, indicating its therapeutic potential in overcoming resistance to ICI therapy (Clift et al., 2019).

Another ECM barrier targeting approach involves lysyl oxidase (LOX) family proteins which play a central role in tumor fibrosis and metastasis through the promotion of EMT and collagen rearrangement within the TME. Specifically, lysyl oxidase-like 2 (LOXL2) has been shown to potentiate angiogenesis and activate FAK/Src pathway, thereby accelerating tumor progression (Liew et al., 2017; Li et al., 2024). Elevated LOX expression correlates with tumor metastasis and reduced OS (Jia et al., 2022). In pre-clinical cancer models, LOX enzyme inhibitor beta-aminopropionitrile (BAPN) led to ECM remodeling, including reduced overall matrix stiffness and reorientation of collagen fibers into a more dispersed organization. These changes facilitate CD8^+^ T cell infiltration into the tumor and stroma region, thereby significantly enhancing the efficacy of anti-PD-1 therapy, as evidenced by increased expression of effector cytokines (Nicolas-Boluda et al., 2021). Likewise, ellagic acid (EA), a LOXL2 inhibitor attenuates collagen crosslinking and deposition, resulting in decrease frequencies of PD1^+^ TIM^+^ CD8^+^ T cell and increased CD8^+^ T cell infiltration. LOXL2 inhibition has been shown to enhance anti-PD-L1 and anti-PD-1 antibodies efficacy, concomitant with an increased overall abundance of CD8^+^ TILs (Peng et al., 2020).

Hypoxia is a hallmark of the rapidly proliferating TME, due to elevated oxygen consumption and insufficient vascularization. Under hypoxic conditions, hypoxia-inducible factors (HIF) evade proteasomal degradation and drive pathways that support angiogenesis and metastasis (Wicks & Semenza, 2022). PX-478, a HIF-1α inhibitor (S-2-amino-3-[4′-N,N,-bis(chloroethyl)amino] phenyl propionic acid N-oxide dihydrochloride) increases CD8^+^ T cell infiltration and granzyme B production when administered in combination with anti-PD-1, resulting in tumor volume reduction. These effects are likely mediated *via* suppression of hypoxia-driven EMT and improvement of anti-PD-1 efficacy (Luo et al., 2022).

Finally, platelet-derived growth factor (PDGF) which is abundantly expressed in stroma cells, contributes to tumor progression *via* activation of cancer associated fibroblast (CAF) and infiltration of TAM, ECM remodeling and metastasis (Li et al., 2022c). Regorafanib, a multi-kinase inhibitor targeting PDGF receptor (PDGFR)α and PDGFRβ, modulates the tumor stroma by targeting CAF and alters the immunosuppressive environment by attenuating immunosuppressive neutrophils and reducing stromal size. Therefore, a combination of PDGFR inhibitor with anti-PD-1 may overcome resistance in fibrotic tumors and increase infiltration of IFN-γ^+^ cytotoxic T cells (Akiyama et al., 2023). Together, all these findings highlight that targeting ECM stiffening and its associated pathways not only alleviates physical and biochemical barriers to immune cell infiltration but also reprograms the TME to improve the efficacy of ICIs and other anti-cancer therapies.

## Challenges and Future Perspectives

Despite the well-established prognostic value of TILs in solid tumors, their therapeutic exploitation remains challenging. Key barriers include inadequate immune cell infiltration, dysregulated chemokine signaling, stromal and extracellular matrix–mediated physical constraints, and persistent T cell exhaustion driven by chronic antigen exposure and immunosuppressive cell populations within the TME. While cytokine-based therapies were among the earliest FDA-approved immunotherapies, followed by immune checkpoint inhibitors and oncolytic viruses, however, their clinical applications are often limited by toxicity and delivery constraints. Cytokine therapies, in particular, frequently require high doses to achieve efficacy, resulting in significant systemic toxicity and necessitating careful risk-benefit evaluation. Similarly, immune checkpoint inhibitors and other immunotherapeutic approaches can induce immune-related adverse events, especially at higher doses, requiring close patient monitoring. In addition, many therapeutic agents suffer from poor tumor penetration and limited targeting specificity, leading to off-target effects and reduced therapeutic indices. Even though cellular immunotherapies have achieved substantial success in hematological malignancies, their translation to solid tumors remains limited by tumor heterogeneity, immune evasion, and low immunogenicity, underscoring the need for strategies that enhance TIL survival, functional persistence, and tumor-specific activity.

Future advances in TIL-based immunotherapy are likely to emerge from integrative, mechanism-driven strategies that concurrently address immune dysfunction and the structural complexity of the TME. Rather than relying on single-modality interventions, next-generation therapies will increasingly combine immune cell engineering to enhance tumor homing and persistence, localized cytokine modulation to amplify antitumor immunity while limiting systemic toxicity, immune checkpoint blockade to reinvigorate exhausted effector cells, and targeted remodeling of the ECM to overcome physical barriers to infiltration. The clinical translation of tumor-homing platforms with reduced dosing requirements and improved safety profiles may redefine how future immunotherapies are delivered in solid tumors. In parallel, advances in tumor profiling and systems immunology are expected to yield predictive biomarkers that enable more precise patient stratification and less restrictive eligibility criteria. Together, these innovations point towards a future in which rationally designed, combinatorial immunotherapies achieve durable TIL-mediated tumor control across a broader spectrum of solid malignancies.

## Conclusions

Overall, the functional heterogeneity of TILs underpins their diverse roles in tumor progression and immune regulation. Following migration from HEV into the TME, the balance and crosstalk among TIL subsets can promote either antitumor immunity or tumor progression, depending on tumor context. Immune cell infiltration and effector function are frequently limited by T cell exhaustion, immunosuppressive cytokine signaling, and dysregulated chemokine axes, contributing to variable and often suboptimal responses to immunotherapy. Although immune checkpoint inhibitors, adoptive cell therapies, and oncolytic viruses have advanced cancer treatment, their efficacy in solid tumors remain constrained by insufficient immune infiltration, functional impairment, and treatment-associated toxicities. Consequently, combination-based therapeutic strategies that target both immune dysfunction and the TME are increasingly pursued to overcome these barriers and improve immunotherapeutic efficacy in solid malignancies.

## Additional Information and Declarations

### Data Availability

The following information was supplied regarding data availability: This is a literature review.
